# Biomarkers in Spinocerebellar Ataxias

**DOI:** 10.1007/s12311-025-01856-5

**Published:** 2025-05-24

**Authors:** Thomas Klockgether, Marcus Grobe-Einsler, Jennifer Faber

**Affiliations:** 1 German Center for Neurodegenerative Diseases (DZNE), Venusberg-Campus 1, 53127 Bonn, Germany; 2 Department of Neurology, University Hospital Düsseldorf, Düsseldorf, Germany; 3 Department of Parkinson’s Disease, Sleep and Movement Disorders, University Hospital Bonn, Bonn, Germany

**Keywords:** Biomarker, Clinical trial, Digital assessment, Magnetic resonance imaging, Neurofilament light chain

## Abstract

Biomarkers are defined as measures that indicate biological processes and responses to interventions. Spinocerebellar ataxias (SCAs) are autosomal dominantly inherited, progressive diseases. As targeted therapies for SCAs are being developed, there is a great need for biomarkers for use in clinical trials. Molecular genetic tests are firmly established as diagnostic biomarkers for SCAs. Biomarkers that monitor disease progression are needed in clinical trials that aim at slowing disease progression. Magnetic resonance imaging (MRI) volume measures and– in SCA2 - saccadic velocity are promising candidates, as they have been shown to decrease over time with larger sensitivity than clinical scales. Prognostic biomarkers indicate the likelihood of progression or a future clinical event. Potential candidates are CAG repeat length, blood neurofilament light chain (NfL) concentrations, MRI volume measures, magnetic resonance spectroscopic (MRS) metabolites, digital measures of gait variability and– in SCA2– sensory nerve amplitudes. Response biomarkers, which are capable of detecting a response to an intervention, are essential for interventional trials. In gene silencing trials, the concentrations of the proteins encoded by the targeted genes serve as response biomarkers. To date, assays for expanded ATXN3 are available. NfL has the potential to serve as a response marker across all SCA subtypes, as it is assumed to indicate ongoing neurodegeneration, but available data are yet insufficient. Although development and validation of biomarkers for SCAs are rapidly evolving, there is an urgent need for further, longitudinal, multimodal studies.

Although there is a broad consensus in what a biomarker is, there is no single, generally valid definition [1]. According to the Food and Drug Administration (FDA), a biomarker is defined as a “characteristic that is measured as an indicator of normal biological processes, pathogenic processes, or biological responses to an exposure or intervention, including therapeutic interventions”. In the FDA definition, biomarkers are clearly delineated from clinical outcome assessments (COA), which measure, how an individual feels, functions, or survives [2, 3]. Another definition published in a review article characterizes a biomarker as “a biological observation that substitutes for and ideally predicts a clinically relevant endpoint or intermediate outcome that is more difficult to observe” [1]. Although basically consistent with the FDA definition, this definition emphasizes the advantage of biomarkers of being simpler and easier accessible than final clinical endpoints, and that they can be analyzed repeatedly and over shorter periods of time [1].

Spinocerebellar ataxia (SCAs) are autosomal dominantly inherited, progressive diseases. Genetically, they fall into two major groups: those caused by dynamic repeat expansion mutations (repeat expansion SCAs) and those caused by non-repeat mutations. To date, at least 50 different SCAs have been genetically identified [4–6]. While biomarkers are routinely used in numerous clinical indications, their actual value for SCAs lies in their application for clinical trials [7].

A prerequisite for the biological significance of biomarkers is their relation to certain pathophysiological events, which lead to manifestation of the disease. Even in monogenic diseases, as the SCAs, there is not a single pathophysiological pathway, but rather parallel and additive processes, which finally lead to ataxia [4]. Biomarkers can thus be classified by the pathophysiological event that they reflect. They are also classified according to their intended use. A widely used classification developed by an FDA-NIH working group defined the following categories: diagnostic biomarker, monitoring biomarker, predictive biomarker, prognostic biomarker, response biomarker, safety biomarker, and susceptibility/risk biomarker. Response biomarkers are further subdivided into pharmacodynamic and surrogate endpoint biomarkers [2, 3]. Complete definitions are given in Table 1. These categories overlap and are not mutually exclusive. E.g., a monitoring biomarker can be used in a clinical trial of an intervention that is hypothesized to slow down disease progression as a response biomarker, but simultaneously as a safety biomarker in the case that the intervention has undesired effects that accelerate progression. Finally, biomarkers are classified by the nature of the measurement. Common categories, which are relevant for SCAs, include genetic, biochemical, imaging, digital, and electrophysiological biomarkers [1, 2].

**Table 1:** Biomarker categories according to BEST (Biomarkers, endpoints, and other Tools) resource [2]

Biomarker category	Definition
Diagnostic	Biomarker used to detect or confirm presence of a disease or condition of interest or to identify individuals with a subtype of the disease
Monitoring	Biomarker measured repeatedly for assessing status of a disease or medical condition or for evidence of exposure to (or effect of) a medical product or an environmental agent
Predictive	Biomarker used to identify individuals who are more likely than similar individuals without the biomarker to experience a favourable or unfavourable effect from exposure to a medical product or an environmental agent
Prognostic	Biomarker used to identify likelihood of a clinical event, disease recurrence or progression in patients who have the disease or medical condition of interest
Response	Biomarker used to show that a biological response, potentially beneficial or harmful, has occurred in an individual who has been exposed to a medical product or an environmental agent Subcategory pharmacodynamic biomarker: Biomarker that indicates biologic activity of a medical product or environmental agent Subcategory surrogate endpoint biomarker: Biomarker that is an endpoint used in clinical trials as a substitute for a direct measure of how a patient feels, functions, or survive
Safety	Biomarker measured before or after an exposure to a medical product or an environmental agent to indicate the likelihood, presence, or extent of toxicity as an adverse effect
Susceptibility/risk	Biomarker that indicates the potential for developing a disease or medical condition in an individual who does not currently have clinically apparent disease or the medical condition

Biomarkers need to be adequately validated before they are used in clinical trials. Validation requires to establish that the respective biomarker measures what is intended to be measured (analytical validation), and that it has the ability to predict or measure the relevant clinical concept (clinical validation) [2]. Depending on the type of biomarker, different clinical data are required for validation. Cross-sectional studies can be used to validate diagnostic biomarker, whereas they are not sufficient to validate other biomarkers. Monitoring, prognostic, and susceptibility biomarkers require longitudinal studies, predictive, pharmacodynamic, and safety biomarkers interventional studies (Fig. 1). To facilitate biomarker validation, the FDA has established a biomarker qualification program, and the European Medicines Agency offers advice for biomarker validation.

**Fig. 1:**
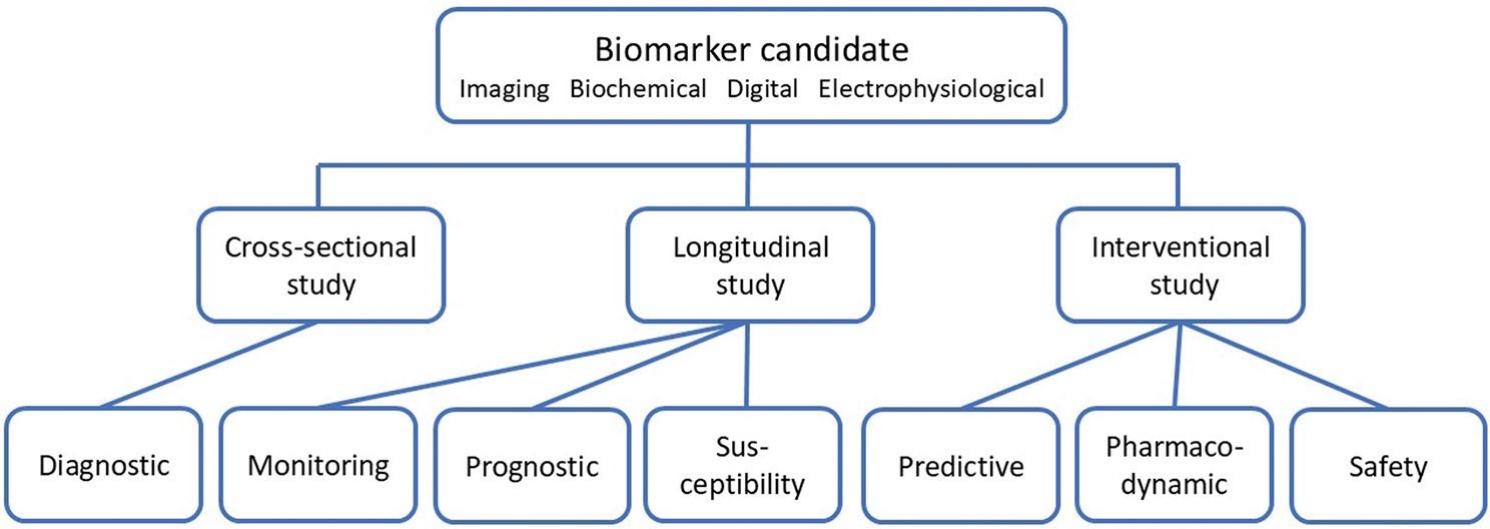
Clinical validation of biomarkers. Biomarker candidates are shown in the top row. Clinical studies are needed for their validation. The different types of studies, which serve to obtain the clinical validation data, are shown in the middle row. The bottom row gives the different types of biomarkers. The connecting lines indicate, which studies are needed for the clinical validation of the various markers

In this article, we will review recent discoveries on biomarkers for SCAs with special emphasis on polyglutamine SCAs, for which most data are available. Biomarker studies of other SCAs are widely lacking. Specifically, there a no studies that systematically compare biomarker findings of polyglutamine SCAs with other SCAs. As cross-sectional studies are of limited use to assess the clinical relevance of potential biomarkers, and as there are only very few interventional studies, we have primarily considered longitudinal studies. We have structured the article according to the nature of the measurement, i.e. genetic, biochemical, imaging, digital, and electrophysiological. For each potential biomarker, we will discuss the corresponding pathophysiological event, the extent and quality of clinical validation to date, and the purpose, for which it can be used.

## Search Strategy

We searched Medline and ISI Web of Science for reports published before Mar 31, 2025, with the search terms [“spinocerebellar ataxia” AND “biomarker” OR “neurofilament light chain (NfL)” OR “MRI“ OR “digital outcome“ AND “prospective“ AND “electrophysiological” OR “follow-up“ OR “longitudinal“]. In addition, we used our own files of research articles.

## Genetic Biomarkers

For the diagnosis of SCAs, molecular genetic tests provide highly reliable information on the presence or absence of the disease-causing mutation. They are thus to be regarded as diagnostic biomarkers. Nevertheless, we will not consider them further in this review, as they are firmly established and routinely used. For more details of molecular genetic testing, we refer to guidelines and a recent review article [8, 9].

In the repeat expansion SCAs, the length of the expanded repeat is associated to varying degrees with the age of onset and the rate of disease progression and may thus be considered as a prognostic biomarker. Most data are available for the polyglutamine SCAs, which are caused by translated, expanded CAG repeats. In a study that included 802 patients with either SCA1, SCA2, SCA3, SCA6, or SCA7, repeat length explained 44.3 to 74.9% of the variance in age at onset [10]. Based on data of two large European SCA cohorts, regression models that predict the age of onset in SCA1, SCA2, SCA3, and SCA6 were developed. Apart from the length of the expanded allele, these models also take into account the actual age and– depending on the SCA subtype– the length of the normal allele [11]. In a large cohort of Chinese SCA3 patients, a logistic survival model using repeat length and actual age as inputs provided the best prediction of the age of onset [12]. The authors of a meta-analysis of genetic risk factors for modulation of age at onset in SCA3 reported earlier age of onset in non-Portuguese Europeans than in Portuguese/South Brazilians with similar CAG repeat lengths underlining the importance of population-specific factors [13]. In SCA27b, which is caused by an intronic GAA repeat expansion of the *FGF14* gene, the relationship between repeat length and age of onset has not yet been finally established [5].

In polyglutamine SCAs, greater length of the expanded CAG repeat is potentially associated with faster ataxia progression. This has been demonstrated for SCA1 [14, 15], SCA2 [16], and SCA3 [17–19]. However, the effect of the repeat length on the rate of progression is generally weak.

## Biochemical Biomarkers

In polyglutamine SCAs, the abnormally elongated proteins encoded by the mutated genes are considered to be the major cause of neurodegeneration. Correspondingly, gene silencing is pursued as a promising therapeutic approach. In trials that investigate gene silencing, sensitive assays that measure the concentration of the corresponding proteins are required as pharmacodynamic biomarkers. While two assay for expanded ATXN3 have been published [20, 21], reports on assays of non-expanded ATXN3 and the corresponding proteins of other polyglutamine SCAs are lacking. In a large longitudinal study of SCA3 mutation carriers, mutant ATXN3 concentrations were constant throughout the entire disease course without major changes over time. Further, mutant ATXN3 was not identified as a predictor of ataxia progression [22].

Neurofilament light chain (NfL) is a biomarker for axonal damage in neurological disorders affecting the central and peripheral nervous system [23]. Increased NfL blood concentrations have been reported in ataxic SCA1, SCA2, SCA3, SCA7, and SCA8 patients [14, 24–33], as well as in SCA1, SCA2, SCA3, and SCA7 pre-ataxic mutation carriers. In these studies, NfL concentrations of pre-ataxic mutation carriers were in an intermediate range between healthy controls and ataxic patients [24, 27, 29–31]. In a study comparing different SCA subtypes, SCA3 had the highest NfL plasma concentrations [26].

NfL plasma levels modestly increased in a 1-year follow-up study of 34 SCA1 patients [14]. Modelling of longitudinal NfL data of a large European cohort of SCA3 mutation carriers, showed a steady increase throughout the disease course with an onset of abnormality of 21.5 years before clinical manifestation [22]. In both studies, responsiveness of NfL was lower than that of the standard clinical outcome, the Scale for the Assessment and Rating of Ataxia (SARA) and of MRI volume markers [14, 22]. Other longitudinal studies in SCA1, SCA2, SCA3, and SCA7 with follow-up periods of 1.0 to 2.7 years did not find increases of NfL blood concentrations [28, 29, 33]. There are only limited data related to the prognostic value of NfL. In SCA1 mutation carriers, time to conversion to manifest ataxia was shorter in individuals with high NfL serum concentrations [28]. In SCA2, higher NfL concentrations predicted cerebellar volume loss [29]. In contrast, NfL was not identified as a predictor of ataxia progression in SCA1 and SCA3 [14, 22]. An overview of NfL studies in SCAs is given Table 2.

**Table 2:** Overview of NfL studies

Study	Subtype	Ataxic	Pre-ataxic	Longitudinal	Prognostic value
Li et al. 2019 [32]	SCA3	increased	not studied	not studied	not studied
Wilke et al. 2020 [30]	SCA3	increased	increased	not studied	not studied
Peng et al. 2020 [31]	SCA3	increased	increased	not studied	not studied
Coarelli et al. 2021 [29]	SCA1	increased	increased	no increase	prediction of volume loss
Coarelli et al. 2021 [29]	SCA2, SCA3, SCA7	increased	not studied	no increase	not studied
Wilke et al. 2022 [28]	SCA1	increased	increased	no increase	conversion
Garcia-Moreno et al. [27]	SCA3	increased	increased	not studied	not studied
Tezenas et al. 2023 [25]	SCA1, SCA3	increased	increased	not studied	not studied
Shen et al. 2023 [26]	SCA2, SCA3, SCA8	increased	increased	not studied	not studied
Faber et al. 2024 [24]	SCA3	increased	increased	not studied	not studied
van Prooije [14]	SCA1	increased	increased	modest increase	no prediction of progression
Coarelli et al. 2024 [33]	SCA2, SCA7	increased	increased	no increase	not studied
Berger et al. 2025 [22]	SCA3	increased	increased	modest increase	no prediction of progression

There are numerous cross-sectional studies reporting abnormalities of biochemical markers in SCAs. Some of the markers were shown to be abnormal already in the pre-ataxic stage, for others, correlations with disease duration and clinical scores were shown. Although some of them hold promise for further development, none of them has yet taken the necessary validation steps to be seriously considered as biomarkers in the strict sense defined above [1, 2]. In particular, there is a complete lack of longitudinal studies of these markers.

Concentrations of CSF glial fibrillary acidic protein (GFAP), an astrocytic marker, were increased in SCA1, whereas blood concentrations were not elevated in SCA1, SCA2, and SCA3 [14, 26, 27]. Plasma ubiquitin carboxy-terminal hydrolase L1 (UCHL1), a neuronal protein involved in proteasomal degradation, was not increased in SCA3 [27]. Total tau was increased in a subgroup of SCA3 mutation carriers [27], whereas phospho-tau was not increased in mixed group of SCA patients [26]. Serum cytokine levels were not altered in a group 79 SCA3 mutation carriers [34]. In SCA2, SCA3, and SCA7, blood markers indicating oxidative stress were increased [35–37]. In SCA2, markers of peripheral inflammation including the neutrophil-to-lymphocyte ratio and the platelet-to-lymphocyte ratio were increased [38]. In SCA3, peripheral blood lymphocyte counts were decreased [39]. miRNAs have been implicated in the pathogenesis of SCAs, particularly in SCA1 and SCA3 [40]. A cross-sectional study of SCA3 serum samples showed dysregulation of miR-25, miR-125b, miR-29a, and miR-34b [41]. In SCA7, a set of four miRNAs discriminated between patients and healthy controls [42].

## Imaging Biomarkers

SCAs are characterized by progressive brain and spinal cord tissue loss that can be studied by structural magnetic resonance imaging (MRI). MRI metrics that appropriately reflect tissue loss include brain regional volumes and spinal cord cross-sectional areas. Several studies in polyglutamine SCAs consistently found that volumes of the cerebellum, brainstem, or basal ganglia decreased over time with effect sizes exceeding that of SARA [14, 22, 43–46]. In contrast, spinal cord cross-sectional areas did not significantly decrease in SCA1 and SCA3 [45, 46]. Pons volume had the highest responsiveness of all volumes in SCA1 [14, 43, 44], SCA3 [22], and SCA7 patients [44], and SCA3 pre-ataxic mutation carriers [22]. In SCA2 patients, cerebellum and brainstem [44, 47], in SCA2 pre-ataxic mutation carriers had the highest responsiveness [47]. In SCA1, pons and cerebellar volume, in SCA3, medulla oblongata were predictors of SARA progression [14, 22].

Diffusion MRI measures provide information on microstructural integrity of white matter tracts. In SCA1 and SCA3 mutation carriers, microstructural abnormalities of the cerebellar peduncles belong to the earliest MRI abnormalities occurring before ataxia onset [48]. In a 6-month longitudinal study of early SCA1 and SCA3, diffusion measures of the middle cerebellar peduncle and corona radiata deteriorated [46]. Mean diffusivity of the left inferior cerebellar peduncle and right medial lemniscus increased within one year in a cohort of 28 SCA3 patients [49]. In a large, longitudinal study of pre-ataxic and ataxic SCA3 mutation carriers, diffusion measures of the inferior and superior cerebellar peduncles indicated progressive microstructural abnormalities, but were less responsive than MRI volume measures [22].

Magnetic resonance spectroscopy (MRS) allows to study metabolic changes of brain tissue. Among the various MRS metabolites, inositol (Ins) is a presumed marker of glial activation and N-acetylaspartate (NAA) of neuronal integrity. In a 1-year longitudinal study of 34 SCA1 individuals, pontine Ins concentrations increased, while total NAA/Ins ratio decreased suggesting progressive neuronal loss with glial activation [14]. Another longitudinal MRS study, however, failed to detect significant changes of metabolite concentrations in SCA1 and SCA3 [46]. Lower baseline levels of NAA and glutamate in the cerebellar white matter were associated with faster ataxia progression in SCA1 [14]. An overview of longitudinal MRI biomarker studies is given in Table 3.

**Table 3:** Overview of longitudinal MRI studies

Study	Subtype	Volume measure		DTI measure		MRS metabolite	
		Sensitivity	Prediction	Sensitivity	Prediction	Sensitivity	Prediction
Reetz et al. 2013 [43]	SCA1	Pons	not studied	not studied	not studied	not studied	not studied
	SCA3	Caudate	not studied	not studied	not studied	not studied	not studied
	SCA6	Caudate	not studied	not studied	not studied	not studied	not studied
Adanyeguh et al. 2018 [44]	SCA1	Pons	not studied	not studied	not studied	not studied	not studied
	SCA2	Cerebellum	not studied	not studied	not studied	not studied	not studied
	SCA3	Pons	not studied	not studied	not studied	not studied	not studied
	SCA7	Pons	not studied	not studied	not studied	not studied	not studied
Piccinin et al. 2020 [45]	SCA3	Cerebellum (Lobule X, Crus II)	not studied	MD (CST, ICP, SCP) RD (CST, ICP, SCP)	not studied	not studied	not studied
Nigri et al. 2020 [47]	SCA2	Brainstem	not studied	not studied	not studied	not studied	not studied
De Oliveira et al. 2023	SCA3 (pre-ataxic)	no progression	not studied	FA (ML)	not studied	not studied	not studied
Rezende et al. 2024 [46]	SCA1	no progression	not studied	FA (MCP, CR) RD (MCP, CR)	not studied	not studied	not studied
	SCA3	Cerebellum	not studied	FA (MCP, CR) RD (MCP, CR)	not studied	no change	no change
van Prooije et al. 2024 [14]	SCA1	Pons	Cerebellum	not studied	not studied	Ins, NAA/Ins ratio (Pons)	NAA/glutamate (CWM)
Berger et al. 2025 [22]	SCA3	Pons	Medulla oblongata	FA (ICP) RD (ICP)	no effect	not studied	not studied
Tang et al. 2025 [49]	SCA3	not studied	not studied	MD (ICP, ML)	not studied	not studied	not studied

Abbreviations: CR– corono radiata, CST– corticospinal tract, CWM– cerebellar white matter, DTI– diffusion tensor imaging, FA– fractional anisotropy, ICP– inferior cerebellar peduncle, Ins– inositol, MCP– middle cerebellar peduncle, MD– mean diffusivity, ML– medial lemniscus, MRI– magnetic resonance imaging, MRS– magnetic resonance spectroscopy, NAA– N-acetylaspartate - RD– radial diffusivity

## Digital Biomarkers

A digital biomarker is defined as a characteristic or set of characteristics, collected from digital health technologies, that is measured as an indicator of normal biological processes, pathogenic processes, or responses to an exposure or intervention, including therapeutic interventions. Digital biomarkers need to be delineated from COAs using digital technology. Thus, a tapping task on the smartphone that measures functional ability is considered as one type of COA, namely a performance outcome (PerfO), while location and time delays between taps represent digital biomarkers [50]. As there are only few longitudinal studies on digital markers in SCAs, we will also refer to cross-sectional studies.

Digital measures of the temporal and spatial variability of gait parameters are considered as potential biomarkers that indicate the pathogenic processes underlying ataxic gait. Available recording techniques include pose estimations based on video recordings, inertial movement units, accelerometers, gyroscope sensors, and pressure-sensitive walkways. As shown in numerous, cross-sectional studies, digital parameters, such as variability of stride length or joint angles, are able to discriminate not only between ataxic and healthy individuals, but also between pre-ataxic SCA mutation carriers and healthy individuals. For an overview we refer to a recent consensus paper [51]. In addition, increased gait variability was shown to be associated with the history of falls in ataxic patients [52]. In a large study of 333 patients with various neurological gait disorders, the patients’ retrospective fall status was the strongest predictor of falls, but the addition of digital measures of gait variability improved the prediction [53]. In a 1-year longitudinal, multicentric study of 17 SCA3 patients, stride length variation and lateral sway during gait with different speeds were more sensitive to change than SARA [54]. Digital measures of gait variability, such as lateral step deviation worsened within one year, whereas SARA remained stable [55]. In a sensor-based, real-life study of 14 patients with various degenerative cerebellar ataxias, lateral velocity change during turning movements detected significant change in 1-year follow-up [56].

Postural stability has been studied with force plates, inertial sensors and video systems. Cross-sectional studies identified a number of digital measures, such as sway area and velocity, that distinguished SCA patients and pre-ataxic mutation carriers from healthy individuals [51, 57, 58]. As with gait studies, there are almost no longitudinal studies of digital balance measures. In a 4-year longitudinal study, a digital measure of body sway, assessed during standing with eyes closed, significantly increased in SCA1 mutation carriers, but not healthy controls [59].

To digitally assess upper limb function in ataxia, various technical systems have been developed and studied in ataxia patients. Examples are the 15-White Dots APP-Coo-Test [60], an extended version of the Q-motor battery [61], and a composite measure derived from inertial sensors [62]. The latter measure has been studied in a longitudinal study of 27 ataxia patients suffering those with polyglutamine SCAs and was shown to capture disease progression with a sensitivity comparable to clinical scales [63]. Although these approaches are very promising, they do not qualify as digital biomarkers in the narrow sense, as they are rather COAs using digital technology.

Digital measures derived from acoustic speech recordings are potential biomarkers that indicate the pathological processes underlying ataxic speech. The potential of digital speech assessment was demonstrated in a study that developed a digital classification system that correctly predicted clinical rating of speech disturbance in ataxia patients [64]. In pre-ataxic SCA2 mutation carriers, reduced speech agility and speech rate correlated with disease severity and time to ataxia onset [65]. Using a neural network trained for phoneme prediction, the average entropy of vowel tokens predictions (AVE) was shown to be associated with ataxia severity and to capture progression even in absence of measured speech decline [66].

## Electrophysiological Biomarkers

There are extensive electrophysiological studies of SCA mutation carriers, both at the pre-ataxic and ataxic stage. Electrophysiological methods are particularly useful to assess peripheral nerve involvement, nerve conduction in central motor and somatosensory pathways, as well as oculomotor abnormalities. Similar to digital biomarkers, there are only few longitudinal studies that allow a rigorous assessment of the potential of individual electrophysiological measures as biomarkers. For an overview we refer to three review articles [67–69].

The peripheral nervous system is often affected in SCAs, with the sensory part usually more severely affected than the motor part. In many publications, the sensory affection is labelled as sensory neuropathy, although available data suggest that it is rather a sensory ganglionopathy or a mixture of axonal, length-dependent neuropathy and ganglionopathy [70–72]. In a longitudinal study of SCA2 pre-ataxic Cuban mutation carriers, sensory amplitudes of the median nerves were reduced 5 to 8 years before onset compared to non-mutation carriers and continued to decline, as subjects approached the onset of ataxia. The amplitudes declined at a rate comparable to those of non-mutation carriers [73]. Similarly, a pseudolongitudinal study of SCA3 patients found reduced sensory amplitudes of the sural nerve, which declined in parallel with those of healthy controls [74]. The Cuban study also reported a progressive increase in the mean latency of the P40 tibial-nerve somatosensory evoked potentials in pre-ataxic SCA2 mutation carriers [73].

Motor evoked potentials following transcranial magnetic stimulation provide measures of integrity of the corticospinal tract, which is affected to various degrees in SCAs. According to a meta-analysis, central motor conduction is mildly prolonged in SCAs with the difference being more obvious in SCA1 than in SCA2, SCA3, and SCA6 [75, 76]. In a 2 year follow-up study of 33 SCA2 pre-ataxic Cuban mutation carriers, resting motor thresholds and central motor conduction times deteriorated, while they remained stable in controls [77].

Saccadic slowing due to pontine brainstem degeneration is a highly characteristic feature of SCA2. Saccadic velocity is reduced both in pre-ataxic and ataxic mutation carriers, and it is inversely related to the CAG repeat length [78, 79]. In a 5-year longitudinal study of 30 Cuban SCA2 patients, saccadic velocity continuously decreased with progression of the disease. Among various, parameters, peak velocity had the highest responsiveness and exceeded that of SARA [80].

## Potential for Application in Clinical Trials

The value of molecular genetic tests to make a definitive diagnosis of a specific SCA is undisputed. In this sense, molecular genetic tests are perfect diagnostic biomarkers. Correspondingly, there is no need for the development and validation of alternative markers that discriminate between SCA mutation carriers and healthy individuals.

Biomarkers that monitor disease progression of SCAs are needed in clinical trials that aim at slowing disease progression. The key feature of monitoring biomarkers is their ability to detect disease progression, preferably with higher sensitivity than COAs. There is no good candidate among the biochemical biomarkers. The largest amount of data is available for NfL, but longitudinal studies did not reveal substantial NfL increase over time. In contrast, MRI volume measures have been shown to decrease over time in polyglutamine SCAs with larger sensitivity than SARA. A particularly promising candidate is pons volume, which is also able to capture progression in pre-ataxic SCA3 mutation carriers [22]. Specifically for SCA2, peak saccadic velocity is a promising candidate [80]. The available, limited data suggest that diffusion MRI measures and MRS metabolite concentrations are less suitable as MRI volume measures to monitor progression.

Predictive biomarkers that are able to identify individuals who are likely to experience a favorable or unfavorable effect from an intervention are of great practical use. Predictive biomarkers are important for enrichment strategies that aim at enhancing the prospect of success of clinical trials [3]. As an example, stratifying a patient population according its progression rate may allow to select a group of rapid progressors, in whom the efficacy of the studied intervention can be demonstrated more easily. Due to the lack of effective interventions, such biomarkers do not exist for SCAs, but some of the prognostic biomarkers discussed in the next paragraph are potential candidates. Likewise, there are currently no biomarkers that predict unfavorable effect of an intervention.

Other than for predictive biomarkers, there are some candidates for prognostic biomarkers, i.e. markers that indicate the likelihood of a future clinical event or progression. In polyglutamine SCAs, CAG repeat length allows a rough estimation of the time to ataxia onset [10]. In SCA1, blood NfL [28], while in SCA2 sensory nerve amplitudes of the median nerve [73] might be useful to estimate the time to ataxia onset, although both studies were performed in small number of mutation carriers. Another example for potential prognostic biomarkers are digital measures of gait variability that may indicate the risk of falls [52, 53]. CAG repeat length is not only associated with the age at ataxia onset, but also ataxia progression. However, the association is weak which limits the value of repeat length as predictor of progression. Recent work in SCA1 and SCA3 suggested the MRI volume measures and MRS metabolites were associated with faster ataxia progression [14, 22]. To make further progress in validation of prognostic biomarkers for SCAs, there is an obvious need for longitudinal studies, both in patients and pre-ataxic mutation carriers.

Response biomarkers, specifically pharmacodynamic biomarkers, which indicate the biologic activity of an intervention, are essential for the execution of interventional trials. In gene silencing trials in polyglutamine SCAs, the concentrations of the proteins encoded by the mutated genes serve as pharmacodynamic (or alternatively target engagement) biomarkers. To date, assays for expanded ATXN3 are available [20, 21]. There is an urgent need to develop assays for non-expanded ATXN3 and for the proteins encoded by genes associated with polyglutamine SCA subtypes other than SCA3.

While the concentrations of the proteins encoded by the mutated genes are subtype-specific, NfL has the potential to serve as a pharmacodynamic biomarker across all SCA subtypes. NfL originates from neuronal axons, and increased concentrations are assumed to indicate ongoing neurodegeneration [23]. Its utility as a response biomarker in clinical trials in neurodegenerative disease is underlined by the results of the tofersen phase 3 trial in amyotrophic lateral sclerosis due to SOD1 mutations, which led to the approval of tofersen in USA and Europe [81]. In polyglutamine SCAs, blood NfL concentrations are abnormally elevated many years before ataxia onset and remain at these levels with only minor further increase throughout the disease course. An NfL decrease due to a therapeutic intervention would thus indicate a slowing of neurodegeneration [28]. Such an assumption would be strongly supported by data showing that NfL is a predictor of a clinically relevant outcome, such as conversion to manifest ataxia or ataxia progression. Despite the good rationale for the utility of NfL as response marker in trials ins SCAs, there is currently a lack of sufficient, longitudinal data to support this claim.

Due to the lack of therapies, there are currently no safety biomarkers for SCAs to monitor the safety of an intervention. However, such markers might be of great value in upcoming trials in SCAs. NfL may be useful to detect adverse events of intrathecally applied antisense oligonucleotides, such as radiculitis [82], but published data to support this hypothesis are still lacking.

Susceptibility/risk biomarkers that indicate the potential for developing a disease or a medical condition are of minor significance in SCAs, as the disease risk is determined by the gene mutation. Conversion to manifest ataxia can be considered as a medical condition. Biomarkers that have the potential to predict the risk and time of conversion have been discussed above in the context of prognostic biomarkers.

## Conclusions

Development and validation of biomarkers for SCAs are rapidly evolving. Nevertheless, only few biomarkers are firmly established for SCAs. While there is growing evidence for MRI volume measures as biomarkers to monitor progression, there is still a lack of data on sufficiently validated prognostic and response biomarkers for SCAs. Consequently, further, longitudinal, multimodal biomarker studies are needed.

## Data Availability

No datasets were generated or analysed during the current study.

## References

[1] Aronson JK, Ferner RE. Biomarkers-a general review. Curr Protoc Pharmacol. 2017;76:9.23.1–9.23.17 10.1002/cpph.19. 10.1002/cpph.19 28306150

[2] FDA-NIH Biomarker Working Group. BEST (Biomarkers, EndpointS, and other Tools) Resource; 2021. 27010052

[3] Califf RM. Biomarker definitions and their applications. Exp Biol Med (Maywood). 2018;243:213–21. 10.1177/1535370217750088. 29405771 10.1177/1535370217750088 PMC5813875

[4] Klockgether T, Mariotti C, Paulson HL. Spinocerebellar ataxia. Nat Rev Dis Primers. 2019;5:24. 30975995 10.1038/s41572-019-0074-3

[5] Pellerin D, Danzi MC, Wilke C, Renaud M, Fazal S, Dicaire M-J, et al. Deep intronic FGF14 GAA repeat expansion in Late-Onset cerebellar Ataxia. N Engl J Med. 2023;388:128–41. 10.1056/NEJMoa2207406. 36516086 10.1056/NEJMoa2207406 PMC10042577

[6] Figueroa KP, Gross C, Buena-Atienza E, Paul S, Gandelman M, Kakar N, et al. A GGC-repeat expansion in ZFHX3 encoding polyglycine causes spinocerebellar ataxia type 4 and impairs autophagy. Nat Genet. 2024;56:1080–9. 10.1038/s41588-024-01719-5. 38684900 10.1038/s41588-024-01719-5

[7] Ashizawa T, Öz G, Paulson HL. Spinocerebellar ataxias: prospects and challenges for therapy development. Nat Rev Neurol. 2018;14:590–605. 10.1038/s41582-018-0051-6. 30131520 10.1038/s41582-018-0051-6 PMC6469934

[8] Coarelli G, Wirth T, Tranchant C, Koenig M, Durr A, Anheim M. The inherited cerebellar ataxias: an update. J Neurol. 2023;270:208–22. 10.1007/s00415-022-11383-6. 36152050 10.1007/s00415-022-11383-6 PMC9510384

[9] Sequeiros J, Martindale J, Seneca S, Giunti P, Kämäräinen O, Volpini V, et al. EMQN best practice guidelines for molecular genetic testing of SCAs. Eur J Hum Genet. 2010;18:1173–6. 10.1038/ejhg.2010.8. 20179742 10.1038/ejhg.2010.8 PMC2987475

[10] van de Warrenburg BP, Hendriks H, Durr A, van Zuijlen MC, Stevanin G, Camuzat A, et al. Age at onset variance analysis in spinocerebellar ataxias: a study in a Dutch-French cohort. Ann Neurol. 2005;57:505–12. 15747371 10.1002/ana.20424

[11] Tezenas du MS, Durr A, Rakowicz M, Nanetti L, Charles P, Sulek A, et al. Prediction of the age at onset in spinocerebellar ataxia type 1, 2, 3 and 6. J Med Genet. 2014;51:479–86. 24780882 10.1136/jmedgenet-2013-102200 PMC4078703

[12] Peng L, Chen Z, Chen T, Lei L, Long Z, Liu M, et al. Prediction of the age at onset of spinocerebellar Ataxia type 3 with machine learning. Mov Disord. 2021;36:216–24. 10.1002/mds.28311. 32991004 10.1002/mds.28311

[13] de Mattos EP, Kolbe MM, Bielefeldt,Leotti V, Saraiva-Pereira ML, Jardim LB. Genetic risk factors for modulation of age at onset in Machado-Joseph disease/spinocerebellar ataxia type 3: a systematic review and meta-analysis. J Neurol Neurosurg Psychiatry. 2019;90:203–10. 30337442 10.1136/jnnp-2018-319200

[14] van Prooije TH, Kapteijns KCJ, van Asten JJA, IntHout J, Verbeek MM, Scheenen TWJ, van de Warrenburg BP, Multimodal. Longitudinal profiling of SCA1 identifies predictors of disease severity and progression. Ann Neurol. 2024;96:774–87. 10.1002/ana.27032. 39096063 10.1002/ana.27032

[15] Jacobi H, Du Montcel ST, Bauer P, Giunti P, Cook A, Labrum R, et al. Long-term disease progression in spinocerebellar ataxia types 1, 2, 3, and 6: a longitudinal cohort study. Lancet Neurol. 2015;14:1101–8. 26377379 10.1016/S1474-4422(15)00202-1

[16] Klockgether T, Lüdtke R, Kramer B, Abele M, Bürk K, Schols L, et al. The natural history of degenerative ataxia: a retrospective study in 466 patients. Brain. 1998;121:589–600. 9577387 10.1093/brain/121.4.589

[17] Jardim LB, Hauser L, Kieling C, Saute JA, Xavier R, Rieder CR, et al. Progression rate of neurological deficits in a 10-year cohort of SCA3 patients. Cerebellum. 2010;9:419–28. 20467850 10.1007/s12311-010-0179-4

[18] Lin Y-C, Lee Y-C, Hsu T-Y, Liao Y-C, Soong B-W. Comparable progression of spinocerebellar ataxias between Caucasians and Chinese. Parkinsonism Relat Disord. 2019;62:156–62. 10.1016/j.parkreldis.2018.12.023. 30591349 10.1016/j.parkreldis.2018.12.023

[19] Peng L, Peng Y, Chen Z, Wang C, Long Z, Peng H, et al. The progression rate of spinocerebellar ataxia type 3 varies with disease stage. J Transl Med. 2022;20:226. 10.1186/s12967-022-03428-1. 35568848 10.1186/s12967-022-03428-1 PMC9107762

[20] Prudencio M, Garcia-Moreno H, Jansen-West KR, Al-Shaikh RH, Gendron TF, Heckman MG, et al. Toward allele-specific targeting therapy and pharmacodynamic marker for spinocerebellar ataxia type 3. Sci Transl Med. 2020. 10.1126/scitranslmed.abb7086. 33087504 10.1126/scitranslmed.abb7086 PMC7927160

[21] Hübener-Schmid J, Kuhlbrodt K, Peladan J, Faber J, Santana MM, Hengel H, et al. Polyglutamine-Expanded Ataxin-3: A target engagement marker for spinocerebellar Ataxia type 3 in peripheral blood. Mov Disord. 2021;36:2675–81. 10.1002/mds.28749. 34397117 10.1002/mds.28749

[22] Berger M, Garcia-Moreno H, Hübener-Schmid J, Schaprian T, Wegner P, Elter T, et al. Progression of biological markers in spinocerebellar ataxia type 3: analysis of longitudinal data from the ESMI cohort. medRvix. medRxiv 2025.01.30.25321426; doi: https://doi.org/10.1101/2025.01.30.25321426. 10.1016/j.lanepe.2025.101339 PMC12270660 40678042

[23] Gaetani L, Blennow K, Calabresi P, Di Filippo M, Parnetti L, Zetterberg H. Neurofilament light chain as a biomarker in neurological disorders. J Neurol Neurosurg Psychiatry. 2019;90:870–81. 10.1136/jnnp-2018-320106. 30967444 10.1136/jnnp-2018-320106

[24] Faber J, Berger M, Wilke C, Hubener-Schmid J, Schaprian T, Santana MM, et al. Stage-Dependent biomarker changes in spinocerebellar Ataxia type 3. Ann Neurol. 2024;95:400–6. 10.1002/ana.26824. 37962377 10.1002/ana.26824

[25] Du Tezenas Montcel S, Petit E, Olubajo T, Faber J, Lallemant-Dudek P, Bushara K, et al. Baseline clinical and blood biomarkers in patients with preataxic and Early-Stage disease spinocerebellar Ataxia 1 and 3. Neurology. 2023;100:e1836–48. 10.1212/WNL.0000000000207088. 36797067 10.1212/WNL.0000000000207088 PMC10136009

[26] Shen X-N, Wu K-M, Huang Y-Y, Guo Y, Huang S-Y, Zhang Y-R, et al. Systematic assessment of plasma biomarkers in spinocerebellar ataxia. Neurobiol Dis. 2023;181:106112. 10.1016/j.nbd.2023.106112. 37003406 10.1016/j.nbd.2023.106112

[27] Garcia-Moreno H, Prudencio M, Thomas-Black G, Solanky N, Jansen-West KR, Hanna Al-Shaikh R, et al. Tau and neurofilament light-chain as fluid biomarkers in spinocerebellar ataxia type 3. Eur J Neurol. 2022;29:2439–52. 10.1111/ene.15373. 35478426 10.1111/ene.15373 PMC9543545

[28] Wilke C, Mengel D, Schöls L, Hengel H, Rakowicz M, Klockgether T, et al. Levels of neurofilament light at the preataxic and ataxic stages of spinocerebellar Ataxia type 1. Neurology. 2022;98:e1985–96. 10.1212/WNL.0000000000200257. 35264424 10.1212/WNL.0000000000200257 PMC9162044

[29] Coarelli G, Darios F, Petit E, Dorgham K, Adanyeguh I, Petit E, et al. Plasma neurofilament light chain predicts cerebellar atrophy and clinical progression in spinocerebellar ataxia. Neurobiol Dis. 2021;153:105311. 10.1016/j.nbd.2021.105311. 33636389 10.1016/j.nbd.2021.105311

[30] Wilke C, Haas E, Reetz K, Faber J, Garcia-Moreno H, Santana MM, et al. Neurofilaments in spinocerebellar ataxia type 3: blood biomarkers at the preataxic and ataxic stage in humans and mice. EMBO Mol Med. 2020;12:e11803. 10.15252/emmm.201911803. 32510847 10.15252/emmm.201911803 PMC7338806

[31] Peng Y, Zhang Y, Chen Z, Peng H, Wan N, Zhang J, et al. Association of serum neurofilament light and disease severity in patients with spinocerebellar ataxia type 3. Neurology. 2020;95:e2977–87. 32817181 10.1212/WNL.0000000000010671

[32] Li Q-F, Dong Y, Yang L, Xie J-J, Ma Y, Du Y-C, et al. Neurofilament light chain is a promising serum biomarker in spinocerebellar ataxia type 3. Mol Neurodegener. 2019;14:39. 10.1186/s13024-019-0338-0. 31684998 10.1186/s13024-019-0338-0 PMC6829913

[33] Coarelli G, Dubec-Fleury C, Petit E, Sayah S, Fischer C, Nassisi M, et al. Longitudinal changes of clinical, imaging, and fluid biomarkers in preataxic and early ataxic spinocerebellar Ataxia type 2 and 7 carriers. Neurology. 2024;103:e209749. 10.1212/WNL.0000000000209749. 39133883 10.1212/WNL.0000000000209749 PMC11361831

[34] Da Silva Carvalho G, Saute JAM, Haas CB, Torrez VR, Brochier AW, Souza GN, et al. Cytokines in Machado Joseph disease/spinocerebellar Ataxia 3. Cerebellum. 2016;15:518–25. 10.1007/s12311-015-0719-z. 26395908 10.1007/s12311-015-0719-z

[35] Dennis A-G, Almaguer-Mederos LE, Raúl R-A, Roberto R-L, Luis V-P, Dany C-A, et al. Redox imbalance associates with clinical worsening in spinocerebellar Ataxia type 2. Oxid Med Cell Longev. 2021;2021:9875639. 10.1155/2021/9875639. 33688396 10.1155/2021/9875639 PMC7920744

[36] de Assis AM, Saute JAM, Longoni A, Haas CB, Torrez VR, Brochier AW, et al. Peripheral oxidative stress biomarkers in spinocerebellar Ataxia type 3/Machado-Joseph disease. Front Neurol. 2017;8:485. 10.3389/fneur.2017.00485. 28979235 10.3389/fneur.2017.00485 PMC5611390

[37] Torres-Ramos Y, Montoya-Estrada A, Cisneros B, Tercero-Pérez K, León-Reyes G, Leyva-García N, et al. Oxidative stress in spinocerebellar Ataxia type 7 is associated with disease severity. Cerebellum. 2018;17:601–9. 10.1007/s12311-018-0947-0. 29876803 10.1007/s12311-018-0947-0

[38] Vázquez-Mojena Y, Rodríguez-Córdova Y, Dominguez-Barrios Y, León-Arcia K, Miranda-Becerra D, Gonzalez-Zaldivar Y, et al. Peripheral inflammation links with the severity of clinical phenotype in spinocerebellar Ataxia 2. Mov Disord. 2023;38:880–5. 10.1002/mds.29359. 36811296 10.1002/mds.29359

[39] Deng Q, Tang C, Chen Z, Yuan X, Ding Z, Wang C, et al. Decreased peripheral blood lymphocytes in spinocerebellar Ataxia type 3 correlate with disease severity. Mov Disord. 2025. 10.1002/mds.30189. 40207410 10.1002/mds.30189

[40] Viswambharan V, Thanseem I, Vasu MM, Poovathinal SA, Anitha A. MiRNAs as biomarkers of neurodegenerative disorders. Biomark Med. 2017;11:151–67. 10.2217/bmm-2016-0242. 28125293 10.2217/bmm-2016-0242

[41] Shi Y, Huang F, Tang B, Li J, Wang J, Shen L, et al. MicroRNA profiling in the serums of SCA3/MJD patients. Int J Neurosci. 2014;124:97–101. 10.3109/00207454.2013.827679. 23879331 10.3109/00207454.2013.827679

[42] Borgonio-Cuadra VM, Valdez-Vargas C, Romero-Córdoba S, Hidalgo-Miranda A, Tapia-Guerrero Y, Cerecedo-Zapata CM, et al. Wide profiling of Circulating MicroRNAs in spinocerebellar Ataxia type 7. Mol Neurobiol. 2019;56:6106–20. 10.1007/s12035-019-1480-y. 30721448 10.1007/s12035-019-1480-y

[43] Reetz K, Costa AS, Mirzazade S, Lehmann A, Juzek A, Rakowicz M, et al. Genotype-specific patterns of atrophy progression are more sensitive than clinical decline in SCA1, SCA3 and SCA6. Brain. 2013;136:905–17. 23423669 10.1093/brain/aws369

[44] Adanyeguh IM, Perlbarg V, Henry PG, Rinaldi D, Petit E, Valabregue R, et al. Autosomal dominant cerebellar ataxias: imaging biomarkers with high effect sizes. Neuroimage Clin. 2018;19:858–67. 29922574 10.1016/j.nicl.2018.06.011 PMC6005808

[45] Piccinin CC, Rezende TJR, de Paiva JLR, Moysés PC, Martinez ARM, Cendes F, França MC. A 5-Year longitudinal clinical and magnetic resonance imaging study in spinocerebellar Ataxia type 3. Mov Disord. 2020;35:1679–84. 10.1002/mds.28113. 32515873 10.1002/mds.28113

[46] Rezende TJR, Petit E, Park YW, Du Tezenas Montcel S, Joers JM, DuBois JM, et al. Sensitivity of advanced magnetic resonance imaging to progression over six months in early spinocerebellar Ataxia. Mov Disord. 2024. 10.1002/mds.29934. 39056163 10.1002/mds.29934 PMC11490388

[47] Nigri A, Sarro L, Mongelli A, Pinardi C, Porcu L, Castaldo A, et al. Progression of cerebellar atrophy in spinocerebellar Ataxia type 2 gene carriers: A longitudinal MRI study in preclinical and early disease stages. Front Neurol. 2020;11:616419. 10.3389/fneur.2020.616419. 33384659 10.3389/fneur.2020.616419 PMC7770103

[48] Chandrasekaran J, Petit E, Park YW, Du Montcel ST, Joers JM, Deelchand DK, et al. Clinically meaningful magnetic resonance endpoints sensitive to preataxic spinocerebellar Ataxia types 1 and 3. Ann Neurol. 2023;93:686–701. 10.1002/ana.26573. 36511514 10.1002/ana.26573 PMC10261544

[49] Tang J, Xing W, Wang D, Qin Y, Li J, Zhang Y, et al. White matter functional and structural alterations of spinocerebellar ataxia type 3: A longitudinal MRI study. Neuroscience. 2025;567:77–85. 10.1016/j.neuroscience.2024.12.055. 39746644 10.1016/j.neuroscience.2024.12.055

[50] Vasudevan S, Saha A, Tarver ME, Patel B. Digital biomarkers: convergence of digital health technologies and biomarkers. NPJ Digit Med. 2022;5:36. 10.1038/s41746-022-00583-z. 35338234 10.1038/s41746-022-00583-z PMC8956713

[51] Ilg W, Milne S, Schmitz-Hübsch T, Alcock L, Beichert L, Bertini E, et al. Quantitative gait and balance outcomes for Ataxia trials: consensus recommendations by the Ataxia global initiative working group on Digital-Motor biomarkers. Cerebellum. 2024;23:1566–92. 10.1007/s12311-023-01625-2. 37955812 10.1007/s12311-023-01625-2 PMC11269489

[52] Schniepp R, Wuehr M, Schlick C, Huth S, Pradhan C, Dieterich M, et al. Increased gait variability is associated with the history of falls in patients with cerebellar ataxia. J Neurol. 2014;261:213–23. 10.1007/s00415-013-7189-3. 24263407 10.1007/s00415-013-7189-3

[53] Schniepp R, Huppert A, Decker J, Schenkel F, Schlick C, Rasoul A, et al. Fall prediction in neurological gait disorders: differential contributions from clinical assessment, gait analysis, and daily-life mobility monitoring. J Neurol. 2021;268:3421–34. 10.1007/s00415-021-10504-x. 33713194 10.1007/s00415-021-10504-x PMC8357767

[54] Ilg W, Müller B, Faber J, van Gaalen J, Hengel H, Vogt IR, et al. Digital gait biomarkers allow to capture 1-Year longitudinal change in spinocerebellar Ataxia type 3. Mov Disord. 2022;37:2295–301. 10.1002/mds.29206. 36043376 10.1002/mds.29206

[55] Seemann J, Daghsen L, Cazier M, Lamy J-C, Welter M-L, Giese MA, et al. Digital gait measures capture 1-Year progression in Early-Stage spinocerebellar Ataxia type 2. Mov Disord. 2024;39:788–97. 10.1002/mds.29757. 38419144 10.1002/mds.29757

[56] Thierfelder A, Seemann J, John N, Harmuth F, Giese M, Schüle R, et al. Real-Life turning movements capture subtle longitudinal and preataxic changes in cerebellar Ataxia. Mov Disord. 2022. 10.1002/mds.28930. 35067979 10.1002/mds.28930

[57] Velázquez-Pérez L, Rodriguez-Labrada R, González-Garcés Y, Arrufat-Pie E, Torres-Vega R, Medrano-Montero J, et al. Prodromal spinocerebellar Ataxia type 2 subjects have quantifiable gait and postural sway deficits. Mov Disord. 2021;36:471–80. 10.1002/mds.28343. 33107647 10.1002/mds.28343

[58] Shah VV, Muzyka D, Jagodinsky A, McNames J, Casey H, El-Gohary M, et al. Digital measures of postural sway quantify balance deficits in spinocerebellar Ataxia. Mov Disord. 2024;39:663–73. 10.1002/mds.29742. 38357985 10.1002/mds.29742

[59] Nanetti L, Alpini D, Mattei V, Castaldo A, Mongelli A, Brenna G, et al. Stance instability in preclinical SCA1 mutation carriers: A 4-year prospective posturography study. Gait Posture. 2017;57:11–4. 28551466 10.1016/j.gaitpost.2017.05.007

[60] Arcuria G, Marcotulli C, Galasso C, Pierelli F, Casali C. 15-White Dots APP-Coo-Test: a reliable touch-screen application for assessing upper limb movement impairment in patients with cerebellar ataxias. J Neurol. 2019;266:1611–22. 10.1007/s00415-019-09299-9. 30955123 10.1007/s00415-019-09299-9

[61] Hermle D, Schubert R, Barallon P, Ilg W, Schüle R, Reilmann R, et al. Multifeature quantitative motor assessment of upper limb ataxia including drawing and reaching. Ann Clin Transl Neurol. 2024;11:1097–109. 10.1002/acn3.52024. 38590028 10.1002/acn3.52024 PMC11093241

[62] Eklund NM, Ouillon J, Pandey V, Stephen CD, Schmahmann JD, Edgerton J, et al. Real-life ankle submovements and computer mouse use reflect patient-reported function in adult ataxias. Brain Commun. 2023;5:fcad064. 10.1093/braincomms/fcad064. 36993945 10.1093/braincomms/fcad064 PMC10042315

[63] Manohar R, Yang FX, Stephen CD, Schmahmann JD, Eklund NM, Gupta AS. At-home wearables and machine learning capture motor impairment and progression in adult ataxias. medRvix. 2024. 10.1093/brain/awaf154 PMC12493059 40305762

[64] Grobe-Einsler M, Faber J, Taheri A, Kybelka J, Raue J, Volkening J, et al. SARAspeech-Feasibility of automated assessment of ataxic speech disturbance. NPJ Digit Med. 2023;6:43. 10.1038/s41746-023-00787-x. 36927996 10.1038/s41746-023-00787-x PMC10020430

[65] Vogel AP, Magee M, Torres-Vega R, Medrano-Montero J, Cyngler MP, Kruse M, et al. Features of speech and swallowing dysfunction in pre-ataxic spinocerebellar ataxia type 2. Neurology. 2020;95:e194–205. 32527970 10.1212/WNL.0000000000009776

[66] Isaev DY, Vlasova RM, Di Martino JM, Stephen CD, Schmahmann JD, Sapiro G, Gupta AS. Uncertainty of vowel predictions as a digital biomarker for ataxic dysarthria. Cerebellum. 2024;23:459–70. 10.1007/s12311-023-01539-z. 37039956 10.1007/s12311-023-01539-z PMC10826261

[67] Liang L, Chen T, Wu Y. The electrophysiology of spinocerebellar ataxias. Neurophysiol Clin. 2016;46:27–34. 10.1016/j.neucli.2015.12.006. 26947625 10.1016/j.neucli.2015.12.006

[68] Velázquez-Pérez L, Rodríguez-Labrada R, González-Garcés Y, Vázquez-Mojena Y, Pérez-Rodríguez R, Ziemann U. Neurophysiological features in spinocerebellar ataxia type 2: prospects for novel biomarkers. Clin Neurophysiol. 2022;135:1–12. 10.1016/j.clinph.2021.12.005. 34998091 10.1016/j.clinph.2021.12.005

[69] Kim DH, Kim R, Lee JY, Lee KM. Clinical, imaging, and laboratory markers of premanifest spinocerebellar Ataxia 1, 2, 3, and 6: A systematic review. J Clin Neurol. 2021;17:187–99. 10.3988/jcn.2021.17.2.187. 33835738 10.3988/jcn.2021.17.2.187 PMC8053554

[70] van de Warrenburg BPC, Notermans NC, Schelhaas HJ, van Alfen N, Sinke RJ, Knoers NVAM, et al. Peripheral nerve involvement in spinocerebellar ataxias. Arch Neurol. 2004;61:257–61. 10.1001/archneur.61.2.257. 14967775 10.1001/archneur.61.2.257

[71] Pelosi L, Iodice R, Antenora A, Kilfoyle D, Mulroy E, Rodrigues M, et al. Spinocerebellar ataxia type 2-neuronopathy or neuropathy? Muscle Nerve. 2019;60:271–8. 10.1002/mus.26613. 31228263 10.1002/mus.26613

[72] Estrada R, Galarraga J, Orozco G, Nodarse A, Auburger G. Spinocerebellar ataxia 2 (SCA2): morphometric analyses in 11 autopsies. Acta Neuropathol. 1999;97:306–10. 10090679 10.1007/s004010050989

[73] Velazquez-Perez L, Rodriguez-Labrada R, Canales-Ochoa N, Montero JM, Sanchez-Cruz G, Aguilera-Rodriguez R, et al. Progression of early features of spinocerebellar ataxia type 2 in individuals at risk: a longitudinal study. Lancet Neurol. 2014;13:482–9. 24657153 10.1016/S1474-4422(14)70027-4

[74] Klockgether T, Schols L, Abele M, Burk K, Topka H, Andres F, et al. Age related axonal neuropathy in spinocerebellar ataxia type 3/Machado-Joseph disease (SCA3/MJD). J Neurol Neurosurg Psychiatry. 1999;66:222–4. 10071104 10.1136/jnnp.66.2.222 PMC1736227

[75] Tang Z-C, Chen Z, Shi Y-T, Wan L-L, Liu M-J, Hou X, et al. Central motor conduction time in spinocerebellar ataxia: a meta-analysis. Aging. 2020;12:25718–29. 10.18632/aging.104181. 33232267 10.18632/aging.104181 PMC7803510

[76] Jhunjhunwala K, Prashanth DK, Netravathi M, Jain S, Purushottam M, Pal PK. Alterations in cortical excitability and central motor conduction time in spinocerebellar ataxias 1, 2 and 3: a comparative study. Parkinsonism Relat Disord. 2013;19:306–11. 10.1016/j.parkreldis.2012.11.002. 23219306 10.1016/j.parkreldis.2012.11.002

[77] Velazquez-Perez L, Rodriguez-Labrada R, Torres-Vega R, Ortega-Sanchez R, Medrano-Montero J, Gonzalez-Pina R, et al. Progression of corticospinal tract dysfunction in pre-ataxic spinocerebellar ataxia type 2: A two-years follow-up TMS study. Clin Neurophysiol. 2018;129:895–900. 29550649 10.1016/j.clinph.2018.01.066

[78] Velazquez-Perez L, Seifried C, Santos-Falcon N, Abele M, Ziemann U, Almaguer LE, et al. Saccade velocity is controlled by polyglutamine size in spinocerebellar ataxia 2. Ann Neurol. 2004;56:444–7. 15349876 10.1002/ana.20220

[79] Velazquez-Perez L, Seifried C, Abele M, Wirjatijasa F, Rodriguez-Labrada R, Santos-Falcon N, et al. Saccade velocity is reduced in presymptomatic spinocerebellar ataxia type 2. Clin Neurophysiol. 2009;120:632–5. 19201647 10.1016/j.clinph.2008.12.040

[80] Rodríguez-Labrada R, Velázquez-Pérez L, Auburger G, Ziemann U, Canales-Ochoa N, Medrano-Montero J, et al. Spinocerebellar ataxia type 2: measures of saccade changes improve power for clinical trials. Mov Disord. 2016;31:570–8. 10.1002/mds.26532. 26846400 10.1002/mds.26532

[81] Miller TM, Cudkowicz ME, Genge A, Shaw PJ, Sobue G, Bucelli RC, et al. Trial of antisense oligonucleotide Tofersen for SOD1 ALS. N Engl J Med. 2022;387:1099–110. 10.1056/NEJMoa2204705. 36129998 10.1056/NEJMoa2204705

[82] Lovett A, Chary S, Babu S, Bruneteau G, Glass JD, Karlsborg M, et al. Serious neurologic adverse events in Tofersen clinical trials for amyotrophic lateral sclerosis. Muscle Nerve. 2025. 10.1002/mus.28372. 40017137 10.1002/mus.28372 PMC12060635

